# The Effect of *NUDT15*, *TPMT*, *APEX1*, and *ITPA* Genetic Variations on Mercaptopurine Treatment of Pediatric Acute Lymphoblastic Leukemia

**DOI:** 10.3390/children8030224

**Published:** 2021-03-15

**Authors:** Jae Min Lee, Ye Jee Shim, Do-Hoon Kim, Nani Jung, Jung-Sook Ha

**Affiliations:** 1 Department of Pediatrics, Yeungnam University College of Medicine, Daegu 42415, Korea; mopic@hanmail.net; 2 Department of Pediatrics, Keimyung University School of Medicine, Daegu 42601, Korea; yejeeshim@dsmc.or.kr (Y.J.S.); lemon378@hanmail.net (N.J.); 3 Department of Laboratory Medicine, Keimyung University School of Medicine, Daegu 42601, Korea; kdh@dsmc.or.kr

**Keywords:** 6-mercaptopurine, acute lymphoblastic leukemia, *TPMT*, *NUDT15*, *ITPA*, *APEX*

## Abstract

Mercaptopurine (MP) is a commonly used maintenance regimen for childhood acute lymphoblastic leukemia (ALL). However, 6-MP has a narrow therapeutic index, which causes dose-limiting toxicities in hematopoietic tissues. Recent studies reported several candidate pharmacogenetic markers such as TPMT, NUDT15, ITPA, and APEX1, which predict the possibility of 6-MP related toxicities. The aim of this study is to evaluate the effect of major variants of these genes on 6-MP intolerances and toxicities in pediatric acute lymphoblastic leukemia (ALL) patients. A total of 83 pediatric ALL patients were included (56 males and 27 females). The NUDT15 c.415C>T (rs116855232), NUDT15 c.55_56insGAGTCG (rs746071566), ITPA c.94C>A (rs1127354), ITPA c.IVS2+21A>C (rs7270101), APEX c.190A>G (rs2307486), and TPMT variants were analyzed by sanger sequencing. Correlations between indexes of 6-MP-related toxicities or 6-MP intolerance (absolute neutrophil count [ANC] at several time point, days of ANC < 1 × 10^3^/mm^3^, days of ANC < 0.5 × 10^3^/mm^3^, frequency of febrile neutropenia, maximum AST and ALT, 6-MP dose and 6-MP dose intensity during maintenance therapy) and genetic variations were analyzed. The NUDT15 c.415C>T allele carrier showed significantly low 6-MP doses at the final maintenance therapy period than the wild type carrier (*p* = 0.007). The 6-MP dose intensities at the sixth and final maintenance period were also significantly low in NUDT15 c.415C>T carriers (*p* = 0.003 and 0.008, respectively). However, indexes for neutropenia, days of febrile neutropenia, maximum AST, and ALT levels were not associated with the presence of c.415C>T as well as other analyzed variants. When analyzing the effect of the coexistence of NUDT15 c.415C>T and ITPA c.94C>A, no significant differences were found between the NUDT15 c.415C>T carrier and carrier with both variations. The NUDT15 c.415C>T was the most useful marker to predict 6-MP intolerance among analyzed variants in our study population. Although we could not find association of those variants with 6-MP induced toxicities and the synergistic effects of those variants, a well-planed larger scale study would be helpful in clarifying new candidates and their clinical effects.

## 1. Introduction

Acute lymphoblastic leukemia (ALL) is the most common pediatric malignancy with overall survival rate of 80–90%. One of contributors for this high survival rate is the maintenance therapy with 6-mercaptopurine (6-MP) [1]. 

Mercaptopurine, one of thiopurine drugs, is commonly used as a maintenance regimen for childhood ALL and has played an important role in long-term remission. However, 6-MP has a narrow therapeutic index. It then causes dose-limiting toxicities in hematopoietic tissues, resulting in neutropenia or febrile neutropenia. Because these toxicities negatively affect ALL treatment outcomes, there have been attempts to find the risk factors causing these toxicities especially in the pharmacogenetics area [2,3].

Mercaptopurine is converted to the main therapeutic metabolites 6-thioguanine nucleotides (TGNs), comprising thioguanosine-5′-triphosphate (TGTP), thioguanosine-5′-diphosphate (TGDP), and thioguanosine-5′-monophosphate (TGMP) by multi-enzymatic steps [4]. TGNs have wide spectrum immunosuppressive roles by incorporation into RNA or DNA and inhibit DNA synthesis [5,6]. Thiopurine S-methyltransferase (*TPMT*) is the principal enzyme in the regulation of thiopurine metabolism by metabolizing TGNs to inactive, nontoxic compounds. Patients with a nonfunctional variant allele of *TPMT* have lower *TPMT* enzyme activity, and consequently 6-TGN excessively accumulates in hematopoietic tissues and frequently causes hematopoietic toxicity [7,8,9]. These findings lead to the concept of individualized 6-MP dosing according to *TPMT* genotype. However, *TPMT* alone cannot account for the individual-specific differences in 6-MP sensitivity [3], and the frequency of the variant *TPMT* alleles differs between ethnic groups [10]. Although approximately 10% of the Caucasian population carries a nonfunctional sequence variant, its frequency is exceptionally low in Asian populations, which brought limits to the application of the *TPMT* genotype to regulating dose of 6-MP in Asian ALL patients [11]. 

Recently, several other genes related to thiopurine toxicity have been reported. Nucleoside diphosphate-linked moiety X-type motif 15 (*NUDT15*) is a nucleotide diphosphatase that can convert TGTP to TGMP and is thus responsible for the inactivation of thiopurine metabolites [12]. A nonsynonymous rs116855232 *NUDT15* variant (c.415C>T, p.Arg139Cys) has been reported as a strong risk factor for thiopurine-induced leukopenia and 6-MP intolerance in patients with inflammatory bowel disease and pediatric ALL in several reports [12,13,14,15,16,17]. As the frequency of this SNP is ~6–10% of the East Asian population, *NUDT15* has been suggested as a useful alternative marker for *TPMT* variants in East Asians [18]. Other SNPs in *NUDT15* such as p.Arg139His, p.Val18Ile, p.Val18_Val19insGlyVal, p.Arg34Thr, p.Lys35-Glu, p.Gly17_Val18del, p.Met1Thr, and p.Gly47Arg have also been uncovered and are undergoing evaluation [12,13,14,15,16,17].

Inosine triphosphate pyrophosphatase (*ITPA*) is another enzyme involved in 6-MP metabolism. *ITPA* catalyzes the hydrolysis of inosine triphosphate (ITP) to inosine monophosphate (IMP), protecting cells from the accumulation of harmful nucleotides such as ITP and deoxyinosine triphosphate [19]. The most well established deleterious *ITPA* variants associated with 6-MP-related toxicity are rs1127354 (c.94C>A) and rs7270101 (IVS2+21A>C) [20]. *ITPA* enzyme deficiency arising from genetic variants affects 5–7% of Caucasians and Africans, and up to 15% of Asians [21]. Despite the high frequency of these variation, their clinical relevance in the predictive role for 6-MP toxicities is still controversial.

Apurinic/apyrimidinic endonuclease 1 (*APEX1*) is another gene candidate reported recently. The missense variation rs2307486 (c.190A>G) in *APEX1* is strongly related to early onset MP-related neutropenia in pediatric ALL patients [22]. Although human *APEX1* is the major enzyme in the DNA base excision repair pathway, and *APEX1*-deficient cells have been shown to exhibit sensitivity to antimetabolites in an in vivo study [23], its clinical relevance in 6-MP treatment has yet not been evaluated much.

The aim of this study is to identify the frequencies of major polymorphisms of candidate genes, *TPMT*, *NUDT15*, *ITPA*, and *APEX1*, and evaluate whether these variations and combination of them could predict 6-MP intolerance and toxicity during maintenance therapy in pediatric ALL patients.

## 2. Materials and Methods

### 2.1. Patients and Data Collection

This study was conducted according to the tenets of the Declaration of Helsinki with the approval of the institutional ethics committee (IRB No. 2019-11-074 Keimyung University Dongsan Hoispital, 2019-12-0072 Yeungnam Uni-versity Hospital). A total of 83 pediatric ALL patients were analyzed, including 56 males and 27 females. The median age at diagnosis was 8.3 years (range: 1.0–16.2 years). There were 52 patients who were allocated to the standard-risk group, 25 to the high-risk group, and six were in the very high-risk group, in accordance with National Cancer Institute (NCI) risk group classification [24,25]. The median follow-up duration was 9.1 years (range: 0.9–16.6 years) (Table 1).

Maintenance chemotherapy consisted of oral prednisolone (40 mg/m^2^) for five days and intravenous vincristine (1.5 mg/m^2^) every four weeks, daily oral 6-MP (75 mg/m^2^), weekly oral methotrexate (MTX) (15 mg/m^2^), and intrathecal chemotherapy (MTX alone or triple regimen [MTX, cytarabine, and hydrocortisone]) every 12 weeks. The doses of 6-MP and MTX were adjusted according to the complete blood count (CBC) and liver function tests (LFT) by a blood test every two weeks. During the maintenance chemotherapy, the target WBC values were 1.5–3.5 × 10^3^/mm^3^ with ANC < 1.5–2.0 × 10^3^/mm^3^ [26,27]. The medications were interrupted if the patients developed a grade three or greater hepatic toxicity, based on the Common Terminology Criteria for Adverse Events version 5.0 (CTCAE 5.0); if aspartate aminotransferase (AST) or alanine aminotransferase (ALT) > 5.0–20.0 × upper limits of normal (ULN), or for a blood bilirubin > 3.0–10.0 × ULN. The medications were also stopped in cases of grade three or greater hematologic toxicities (ANC < 1.0–0.5 × 10^3^/mm^3^) with a febrile infectious event occurring during maintenance chemotherapy.

Neutropenia is defined as a neutrophil count of 0.5 × 10^3^/mm^3^ or less. For early neutropenia, ANC at 14th day and 28th day were analyzed. Febrile neutropenia is defined as two or more temperature spikes of 38.0 °C or greater at least 1 hour apart, or as a single spike of 38.3 °C or greater, along with a neutrophil count of 0.5 × 10^3^/mm^3^ or less at the time of the fever or a decrease to 0.5 × 10^3^/mm^3^ or less within 48 h of fever onset. Numbers of neutropenia and febrile neutropenia episodes during maintenance therapy were retrospectively collected using patient medical records.

Data of a 6-MP dose and a 6-MP dose intensity (%) was collected on the 13th (2nd maintenance period), and the 61st (6th maintenance period) week during maintenance chemotherapy, as well as at the final maintenance period. The 6-MP dose intensity (%) was calculated as the ratio of the actual prescribed 6-MP dose by physician and the protocol dose. Hepatotoxicity was defined as an ALT or AST level > 200 U/L at any time point during maintenance therapy.

### 2.2. Genotyping

Genotyping for the following polymorphisms from five genes was performed: *NUDT15* c.415C>T (rs116855232), *NUDT15* c.55_56insGAGTCG (rs746071566), *ITPA* c.94C>A (rs1127354), *ITPA* c.IVS2+21A>C (rs7270101), *APEX1* c.190A>G (rs2307486), TPMT*2 c.238G>C (rs1800462), TPMT*3B c.460G>A (rs1800460), TPMT*3C c.719A>G (rs1142345), TPMT*4 c.626-1G>A (rs1800584), and TPMT*3A being the combination of the TPMT*3B and TPMT*3C genotypes.

Genomic DNA was isolated from 150 μL peripheral blood using the QIAamp DNA blood Mini kit from Qiagen (Germantown, MD, USA) and stored at −20 °C until analysis. Amplification was performed using Smart Tag Premix (Solgent Co., Daejeon, Korea). Briefly, 200 ng of DNA template and 1 μL each of forward and reverse primers were added to the PCR premix (20 μL final volume). Sanger sequencing was performed using the following primers: *NUDT15* c.415C>T, forward 5′-CAAGCAAATGCAAAGCATCA reverse 5′-GGCTGAAAGAGTGGGGGATA; *NUDT15* c.55_56insGAGTCG, forward 5′-GCGCTCTCGCTTTGATTTC reverse 5′-GAACGCGCAAGAAAGGAC; *ITPA* c.94C>A and IVS2+21A>C, forward 5′-TAGGAGATGGGCAGCAGAGT reverse 5′-CCTGGAAGCTACCTGGACAA; *APEX1* c.190A>G, forward 5′-CAGTTGGAAACCACCAGCTT reverse 5-CAGAAAACTACGGGCAGGAG; *TPMT* c.238G>C, forward 5′-TCCTGCATGTTCTTTGAAACC reverse 5′-TCGGTGATTGGTTCTTCTGA; *TPMT* c.460G>A, forward 5′-GGGACGCTGCTCATCTTCT reverse 3′-TTCAAACTCATAGAAGTCTAAGCTGAT; *TPMT* c.719A>G and c.626-1G>A, forward 5′-GAATCCCTGATGTCATTCTTCA reverse 5′-CATTACATTTTCAGGCTTTAGCA.

Amplification parameters were an initial denaturation step of 95 °C for 30 s, annealing at 60 °C for 30 s, and extension at 72 °C for 45 s for all reactions. The quality and sizes of PCR products were assessed by electrophoresis in a 1.5% agarose gel. Products were purified and bidirectionally sequenced using a BigDye Terminator v3.1 Cycle Sequencing kit (Applied Biosystems, Foster City, CA, USA) on an ABI 3500xl DNA analyzer (Applied Biosystems, 850 Lincoln Centre Drive, Foster City, CA, USA). Mutations were identified using the Sequencher software (version 5.4.5).

### 2.3. Statistical Analysis

Data was entered and analyzed in SPSS v.26 (IBM Corporation, Armonk, NY, USA). Correlations between the index of 6-MP-related toxicities (absolute neutrophil count [ANC], days of ANC < 1 × 10^3^/mm^3^, days of ANC < 0.5 × 10^3^/mm^3^, frequency of febrile neutropenia, maximum AST and ALT), 6-MP intolerance (6-MP dose and 6-MP dose intensity at second, sixth and final maintenance therapy periods), and genetic variations were analyzed using Mann–Whitney U test. A *p*-value of less than 0.05 was considered statistically significant.

## 3. Results

### 3.1. Patient Characteristics, Genotype Data, and Allele Frequencies 

Most of the enrolled children experienced episodes of neutropenia during the entire period of maintenance therapy. Seventy-six (91.6%) children experienced <1 × 10^3^/mm^3^ neutropenia during at least one day (268.3 ± 224.8 days), and 70 (84.3%) children experienced <0.5 × 10^3^/mm^3^ neutropenia during 136.8 ± 132.0 days. Sixty-one (73.5%) children experienced febrile neutropenia during 6.1 ± 5.3 days. An ANC at the 14th day and 28th day from initiation of maintenance therapy was 708.8 ± 824.1/mm^3^, and 188.6 ± 383.4/mm^3^, respectively. The 6-MP dose was started at 75 mg/m^2^ for all patients and an adjusted dose at the second, sixth, and final maintenance therapy cycles were 51.6 ± 19.1 mg/m^2^, 48.5 ± 16.5 mg/m^2^, and 45.3 ± 15.3 mg/m^2^, respectively. The 6-MP dose intensities were 70.0 ± 24.8%, 66.3 ± 22.5%, and 61.7 ± 21.0%, respectively. The maximum AST, ALT levels during maintenance therapy were 303.9 ± 220.7 IU/L and 636.3 ± 458.6 IU/L, respectively (Table 1). The differences of these indices according to age group (<10, ≥10 years), risk group (intermediate, high, and very high groups), and sex were not found (data not shown).

Genotype frequencies for *NUDT15* c.415C>T, *NUDT15* c.55_56insGAGTCG, *ITPA* c.94C>A, *ITPA* c.IVS2+21A>C, *APEX1* c.190A>G, and *TPMT* are summarized in Table 1. Of the 83 children tested, 46 (55.4%) carried at least one of the sequence variants of them: *NUDT15* c.415C>T, 16 (19.3%); *NUDT15* c.55_56insGAGTCG, 9 (10.8%); *ITPA* c.94C>A, 21 (25.3%); *ITPA* c.IVS2+21A>C, 1 (1.2%); *APEX1* c.190A>G, 9 (10.8%); *TPMT* variant 1*/3*, 1 (1.2%). Eight of the *NUDT15* c.55_56insGAGTCG carriers were accompanied by c.415C>T as diplotype *1/*2, and only one patient carried the sole c.55_56insGAGTCG as diplotype *1/*6 [18]. Nine patients carried more than 2 variants and combinations of them are listed in Table 1. Because of low allele frequency or co-occurrence with c.415C>T, the variant of *TPMT*, *ITPA* IVS2+21A>C and *NUDT15* c.55_56insGAGTCG were excluded in the analysis as an independent pharmacogenetic markers.

### 3.2. Associations between Genetic Polymorphisms and 6-Mercaptopurine Related Toxicities and Intolerances

**Figure 1:**
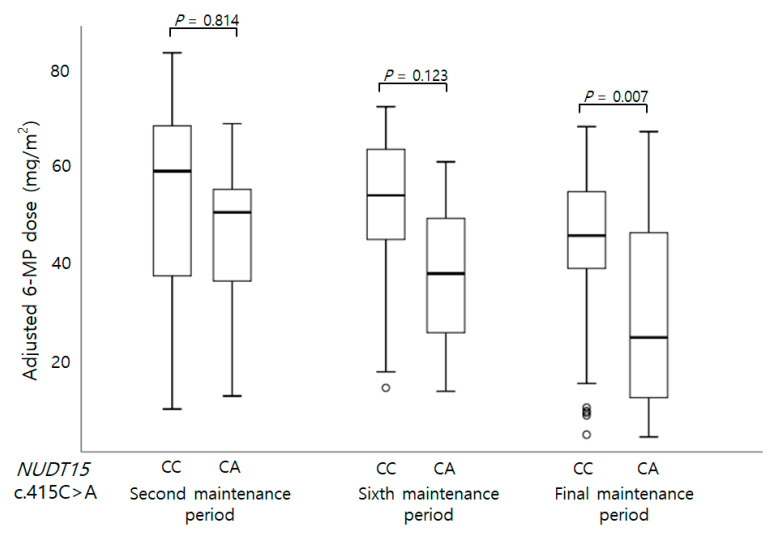
Genotype of *NUDT15* c.415C>A and adjusted 6-MP dose at second, sixth, and final maintenance periods. The adjusted 6-MP dose at final maintenance period was significantly low in patients carrying CA heterozygote than those with wild CC genotype.

**Figure 2:**
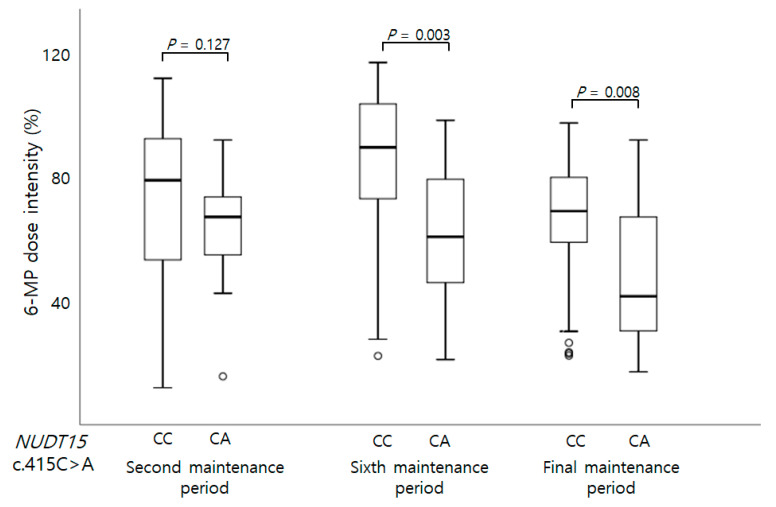
Genotype of *NUDT15* c.415C>A and 6-MP dose intensity (%) at second, sixth, and final maintenance periods. The 6-MP intensity was significantly low in patients carrying CA heterozygote than those with wild genotype at sixth and final maintenance therapy periods.

When the entire maintenance treatment period had been considered, there was no statistically significant associations between the investigated sequence variants and neutropenia (those experienced on days of <5 × 10^3^ and <1 × 10^3^ /mm^3^ neutropenia, days of febrile neutropenia, ANC at 14th and 28th day) (Table 2). The *NUDT15* c.415C>T carriers were likely to have a lower ANC on the 28th day than the wild type, but this was not statistically significant (Table 2). When considering the 6-MP doses at second, sixth, and final maintenance therapy sessions, the *NUDT15* c.415C>T allele carrier showed significantly low 6-MP doses at final maintenance therapies than the wild type carrier (*p* = 0.007 respectively) (Figure 1). The 6-MP dose intensities at the sixth and final maintenance period were also significantly low in *NUDT15* c.415C>T carriers (*p* = 0.003 and 0.008, respectively) (Figure 2). Maximum AST, and ALT levels were not associated with variants. Because of the low number of patients carrying more than two variants, their synergic effect could not be analyzed except for the *NUDT15* c.415C>T and *ITPA* c.94C>A. When analyzing the effect of the coexistence of *NUDT15* c.415C>T and *ITPA* c.94C>A, no 6-MP toxicity differences were found between the *NUDT15* c.415C>T carrier and carrier with both variations (Table S1).

## 4. Discussion

In this study, the authors investigated the effects of major polymorphisms of four genes, *NUDT15*, *ITPA*, *APEX1*, *TPMT* on 6-MP-related toxicities or intolerance. Authors found that *NUDT15* c.415C>T had a lowering effect on 6-MP dose during maintenance therapy in pediatric ALL patients.

As reported in previous studies, the *TPMT* and *ITPA* c.IVS2+21A>C variants were extremely low in our study population [12,28]. Thus, *ITPA* c.IVS2+21A>C and *TPMT* variants were inappropriate for use as a pharmacogenetic marker for thiopurine therapy in Korean ALL patients. The frequencies of *ITPA* c.94C>A and *APEX1* c.190A>G were 25.3% and 10.8%, respectively. This is similar with previous studies reporting 21.9–27.0% [28,29], and 14.1% for Korean population [22].

The *NUDT15* c.415C>T variant was reported to be a major predictive marker for thiopurine-related myelosuppression, particularly in Asian and Hispanic people. Yang et al. identified *NUDT15* c.415C>T as being strongly associated with thiopurine-induced early leukopenia in Crohn’s disease [12]. Tanaka et al. found that leucopenia is more common in *NUDT15* c.415C>T carriers in pediatric ALL patient, and leucopenia results in a significant 6-MP dose reduction [30]. In our study, we collected information about neutropenia, such as the days with <5 × 10^3^ and <1 × 10^3^/mm^3^ neutropenia, days with febrile neutropenia, and we took an ANC on day 14 and 28 of maintenance therapy as early neutropenia index. But we found no significant relationship between genotypes and early neutropenia, neutropenic duration, and febrile neutropenia. However, the doses of 6-MP and dose intensity at the sixth, and final maintenance therapy sessions were significantly low in *NUDT15* c.415C>T carriers. The clinicians regulated the 6-MP doses according to the patients’ neutrophil count, fever event, hepatotoxicity, or patient health status. Therefore, the adjustment of the 6-MP dose could be an indicator for 6-MP intolerance. Yi et al. reported a low 6-MP dose and longer duration of therapy interruption in *NUDT15* c.415C>T carriers [31]. Moreover, Yang et al. found *NUDT15* c.415C>T homozygote carriers were exquisitely sensitive to MP, with a significantly low average dose intensity of 8% in child ALL patients [13]. In addition to *NUDT15* c.415C>T, other variants such as p.Arg139His, p.Val18Ile, p.Val18_Val19insGlyVal, p.Arg34Thr, p.Lys35-Glu, p.Gly17_Val18del, p.Met1Thr, and p.Gly47Arg have also been uncovered. Except for p.Val18_Val19insGlyVal (c.55_56insGAGTCG), other variants are present very infrequently, but these variants also showed the effect of a 74.4–100% loss of nucleotide diphosphatase activity resulting in excessive levels of thiopurine active metabolites and toxicity [16,32]. In particular, the p.G17_V18del variant protein showed extremely low thermostability and was completely void of catalytic activity, thus likely to confer a high risk of thiopurine intolerance [32]. To find out the mechanism of this *NUDT15* variant, a few in vivo studies were conducted. In a recent study of the knock-in mouse model, the *NUDT15*-/- mice experienced severe leukopenia, rapid weight loss, early death, and more bone marrow hypocellularity on 6-MP therapy as compared to the wild type mice [33]. In another study, a clinically relevant dose of MP induced lethal cytopenia in *NUDT15* c.415C>T harboring mice not only in the homozygote variant but also in the heterozygote variant. Even lower-dose MP during long-term administration caused stronger damage than wild type [34]. These in vivo studies support the preemptive *NUDT15* genotype–guided thiopurine dosing to prevent toxicity.

Before *NUDT15*, a variant of *ITPA* was represented as a potential marker of 6-MP sensitivity, especially in populations with a low frequency of the *TPMT* variant. But the clinical relevance of *ITPA* genotyping in 6-MP toxicity prediction is still controversial [35,36,37]. In our study, although the frequency of *ITPA* c.94C>A was sufficiently high, we could not discover a significant relationship with neutropenia or 6-MP dosing. A total of five patients carried both the *ITPA* c.94C>A and *NUDT15* c.415C>T variants. We then compared those patients having only *NUDT15* variant with those who had both the *NUDT15* and *ITPA* variants to analyze their synergistic effects. Although those with both variations were likely to have the lower ANC on the 14th day, we could not find any statistically significant differences of neutropenia or 6-MP dose between either the *NUDT15* carriers or both carriers (Supplementary Table S1). To analyze the synergistic effects of additional other variants, a study of a larger populations is needed.

*APEX1* c.190A>G has relatively recently been proposed as a candidate gene, which has been revealed to have a strong relation with early onset MP-related neutropenia occurring within 28 days as well as a cumulative incidence of MP-related neutropenia [22]. Human *APEX1* is the major enzyme in the DNA base excision repair pathway where its main role is to create a nick in the phosphodiester backbone [23]. The allele frequencies of the variant alleles in c.190A>G were near 0% in Europeans, 0.08% in Africans, and 1.73% in Americans, whereas they were 4.66% in East Asian individuals, according to the 1000 Genomes database [22]. In the previous study of McNeill et al., *APEX1*-deficient cells were shown to exhibit the greatest sensitivity to antimetabolites among various anticancer drugs, and consequently apoptotic cell death was profoundly increased, supporting a role for mutant *APEX1* in thiopurine-induced neutropenia [23]. In our study, although the patient number is small, the frequency of variants was relatively high, 10.8%, and all of them were heterozygote. Although a meaningful effect on 6-MP toxicities was not found in this study, a larger-scale study is needed for a new candidate gene.

The low incidence of the *TPMT* variant in Asian population has brought a discovery of new pharmacogenetic markers predicting 6-MP toxicities, such as *NUDT15*, *ITPA*, and *APEX1*. As new markers have been uncovered, identification of clinical significance and the synergic effect of those markers has been important. In this study, we could not find a distinct predictive role for these variations and their synergistic effect on myelosuppression such as neutropenia, but carriers with *NUDT15* c.415C>T showed lowered 6-MP dose meaning 6-MP intolerance. This limited result might be due to the relatively low numbers of patients or retrospective study design. The sample size of the study is small for assessment of multiple genetic variations and multiple outcomes measurements. Controlling for important covariates is difficult. Because the clinicians already had regulated the 6-MP dose according to the patients’ health state including an ANC, the neutropenic effects of 6-MP might be obscured.

In future studies, a personalized 6-MP dosing plan will be applied in consideration of pharmacokinetics. It is also necessary to validate the effect of *NUDT15*, *TPMT*, *APEX1*, and *ITPA* genetic variations on 6-MP dosing of pediatric ALL through large-scale studies. These studies would help clarify new candidates and increase the number of patients who can benefit from the various pharmacogenetic markers.

## Figures and Tables

**Table 1:** Characteristics and genotype frequencies of pediatric acute lymphoblastic leukemia patients enrolled in this study.

Characteristics	
Total number of patients, *n* (%)	83
Sex, *n* (%)	
Female	27 (32.5)
Male	56 (67.5)
Risk group, *n* (%)	
Standard risk	52 (62.7)
High risk	25 (30.1)
Very high risk	6 (7.2)
Age at diagnosis, median (range)	8.3 (1.0–16.2)
Leukemia type, *n* (%)	
B-ALL	68 (81.9)
T-ALL	8 (9.6)
Mixed-type	4 (4.8)
Unclassified	3 (3.6)
Cytogenetic abnormality, *n* (%)	
Normal	28 (33.7)
Hyperdiploidy	13 (15.7)
t(12;21)(p13;q22); *ETV6-RUNX1*	12 (14.5)
t(4;11)(q21;q23); *MLL-AFF1*	3 (3.6)
t(1;19)(q23;p13.3); *TCF3-PBX1*	3 (3.6)
t(9;22)(q34;q11.2); *BCR-ABL1*	1 (1.2)
Hypodiploidy	1 (1.2)
Other/unknown	22 (26.5)
Neutropenia (mean ± SD)	
ANC at 14th day (/mm^3^)	708.8 ± 824.1
ANC at 28th day (/mm^3^)	188.6 ± 383.4
Days of ANC < 1 × 10^3^/mm^3^ (*n* = 76)	268.3 ± 224.8
Days of ANC < 0.5 × 10^3^/mm^3^ (*n* = 70)	136.8 ± 132.0
Days of febrile neutropenia (*n* = 61)	6.1 ± 5.3
6-MP dose (mg/m^2^, mean ± SD)	
First maintenance	75
Second maintenance	51.6 ± 19.1
Sixth maintenance	48.5 ± 16.5
Final maintenance	45.3 ± 15.3
6-MP dose intensity *	
Second maintenance	70.0 ± 24.8
Sixth maintenance	66.3 ± 22.5
Final maintenance	61.7 ± 21.0
Maximum AST, (IU, mean ± SD)	303.9 ± 220.7
Maximum ALT, (IU, mean ± SD)	636.3 ± 458.6
Genotype, *n* (%)	
*NUDT15* 55_56insGAGTCG (rs746071566)	
Wild	74 (89.2)
55_56insGAGTCG	9 (10.8)
*NUDT15* c.415C>T (rs116855232)	
CC	67 (80.7)
CA	16 (19.3)
*ITPA* c.94C>A (rs1127354)	
CC	62 (74.7)
CA	21 (25.3)
*ITPA* c.IVS2+21A>C (rs7270101)	
AA	82 (98.8)
AC	1 (1.2)
*APEX1* c.190A>G (rs2307486)	
AA	74 (89.2)
AG	9 (10.8)
*TPMT*	
*1/*1	82 (98.8)
*1/*3	1 (1.2)
*NUDT15* c.415C>T + *ITPA* c.94C>A	5 (6.0)
*NUDT15* c.415C>T + *APEX1* c.190A>G	1 (1.2)
*TPMT* *1/*3 + *ITPA* c.94C>A	1 (1.2)
*ITPA* c.94C>A + *APEX1* c.190A>G	2 (2.4)

* 6-MP dose intensity: ratio of actual prescribed 6-MP dose by physician and the protocol dose. Abbreviations: ANC, absolute neutrophil count; 6-MP, 6-mercaptopurine; *NUDT15*, nucleoside diphosphate-linked moiety X-type motif 15; *ITPA*, inosine triphosphate pyrophosphatase; *APEX1*, apurinic/apyrimidinic endonuclease 1; *TPMT*, thiopurine S-methyltransferase.

**Table 2:** Relationships between genotypes of candidate genes and parameters for 6-MP-related toxicities and intolerance.

	*ITPA* rs1127354			*APEX1* rs2307486			*NUDT15* rs116855232		
Charcteristics	Genotype	CC (*n* = 62) CA (*n* = 21)	*p*	Genotype	AA (*n* = 74) AG (*n* = 9)	*p*	Genotype	CC (*n* = 67) CT (*n* = 16)	*p* *
14th day ANC (/mm^3^)	CC	1588.2 ± 1175.2	0.539	AA	1730.8 ± 1577.8	0.070	CC	1688.1 ± 1627.5	0.884
	CA	1850.4 ± 2269.3		AG	814.5 ± 740.1		CT	1421.5 ± 1087.5	
28th day ANC (/mm^3^)	CC	1482.2 ± 1112.5	0.379	AA	1425.9 ± 1169.3	0.677	CC	1561.5 ± 1223.8	0.095
	CA	1274.4 ± 1246.7		AG	1443.8 ± 960.6		CT	892.4 ± 478.8	
Days of ANC < 1 × 10^3^/mm^3^	CC	260.5 ± 228.3	0.540	AA	262.4 ± 223.6	0.451	CC	263.9 ± 224.8	0.564
	CA	290.1 ± 218.3		AG	321.6 ± 244.0		CT	285.9 ± 231.2	
Days of ANC < 0.5 × 10^3^/mm^3^	CC	127.3 ± 130.9	0.317	AA	131.6 ± 128.2	0.377	CC	133.9 ± 130.2	0.527
	CA	163.2 ± 134.6		AG	182.8 ± 165.1		CT	148.3 ± 142.7	
Days of febrile neutropenia	CC	6.00 ± 5.56	0.608	AA	5.9 ± 5.3	0.383	CC	5.7 ± 5.1	0.102
	CA	6.52 ± 4.70		AG	7.5 ± 5.1		CT	7.5 ± 6.0	
6-MP dose at second maintenance period (mg/m^2^)	CC	50.5 ± 7.45	0.319	AA	51.4 ± 19.1	0.837	CC	53.0 ± 19.5	0.814
	CA	54.6 ± 19.0		AG	52.8 ± 19.3		CT	45.6 ± 15.9	
6-MP dose at sixth maintenance period (mg/m^2^)	CC	48.3 ± 16.7	1.000	AA	47.9 ± 17.1	0.615	CC	51.0 ± 15.6	0.123
	CA	48.8 ± 16.4		AG	54.1 ± 6.7		CT	38.1 ± 16.3	
6-MP dose at final maintenance treatment (mg/m^2^)	CC	45.6 ± 15.5	0.912	AA	45.0 ± 15.8	0.928	CC	47.7 ± 13.8	0.007
	CA	44.5 ± 14.9		AG	47.7 ± 8.8		CT	35.4 ± 17.6	
6-MP dose intensity ^†^ at second maintenance period (%)	CC	68.1 ± 25.6	0.235	AA	67.0 ± 24.8	0.877	CC	72.7 ± 24.3	0.127
	CA	75.2 ± 21.9		AG	70.4 ± 25.8		CT	59.2 ± 24.2	
6-MP dose intensity ^†^ at sixth maintenance period (%)	CC	65.5 ± 23.1	0.788	AA	65.6 ± 23.3	0.797	CC	70.6 ± 20.1	0.003
	CA	68.2 ± 21.0		AG	72.1 ± 9.0		CT	49.4 ± 23.6	
6-MP dose intensity ^†^ at final maintenance period (%)	CC	61.6 ± 21.8	0.792	AA	61.5 ± 21.7	0.866	CC	65.9 ± 17.8	0.008
	CA	61.8 ± 18.7		AG	63.6 ± 11.7		CT	45.5 ± 24.5	
Maximum AST	CC	298.3 ± 210.6	0.883	AA	305.7 ± 227.5	0.785	CC	308.1 ± 218.0	0.727
	CA	646.6 ± 485.1		AG	286.8 ± 154.6		CT	286.6 ± 237.5	
Maximum ALT	CC	646.7 ± 485.1	0.974	AA	655.0 ± 474.0	0.255	CC	658.0 ± 478.4	0.427
	CA	606.9 ± 383.6		AG	467.3 ± 240.9		CT	549.1 ± 369.1	

* *p* < 0.05 was considered statistically significant. ^†^ 6-MP dose intensity: the ratio of actual prescribed 6-MP dose by physician and the protocol dose. Abbreviations: ANC, absolute neutrophil count; 6-MP, 6-mercaptopurine; *NUDT15*, nucleoside diphosphate-linked moiety X-type motif 15; *ITPA*, inosine triphosphate pyrophosphatase; *APEX1*, apurinic/apyrimidinic endonuclease 1.

## Data Availability

The data presented in this study are available on request from the corresponding author.

## Supplementary Materials

The following are available online at https://www.mdpi.com/2227-9067/8/3/224/s1. Table S1: Comparison of parameters for 6-MP-related toxicities and intolerance between carriers with *NUDT15* c.415C>T and those with both *NUDT15* c.415C>T and *ITPA* c.94C>A.
