# Supplementary material for: The Effect of NUDT15, TPMT, APEX1, and ITPA Genetic Variations on Mercaptopurine Treatment of Pediatric Acute Lymphoblastic Leukemia
Source: Children (Basel). 2021 Mar 15;8(3):224. doi: 10.3390/children8030224 (PMC7998516; doi:10.3390/children8030224)
Supplement: Supplementary file 1 [file children-08-00224-s001.pdf]

Table S1. Comparison of parameters for 6-MP-related toxicities and intolerance between carriers with *NUDT15* c.415C>T and those with both *NUDT15* c.415C>T and *ITPA* c.94C>A.

Charcteristics	*NUDT15* c.415C>T Only (*N* = 11)	*NUDT15* c.415C>T + *ITPA* c.94C>A (*N* = 5)	*p* *
Neutropenia			
14th day ANC (/mm^3)	1790.6 ± 1106.7	683.4 ± 602.1	0.055
28th day ANC (/mm^3)	940.9 ± 554.1	785.8 ± 267.4	0.913
Days of ANC < 1 × 10^3/mm^3	252.9 ± 189.9	358.6 ± 317.4	0.743
Days of ANC < 0.5 × 10^3/mm^3	118.1 ± 104.6	214.8 ± 202.4	0.743
Days of febrile neutropenia	7.9 ± 6.4	6.6 ± 5.8	0.827
6-MP dose (mg/m^2)			
second maintenance period	41.5 ± 17.3	53.9 ± 9.6	0.165
sixth maintenance period	36.3 ± 16.7	41.9 ± 16.9	0.440
final maintenance treatment	33.6 ± 19.0	39.2 ± 15.8	0.440
6-MP dose intensity † (%)			
second maintenance period	58.9 ± 20.7	71.9 ± 12.9	0.206
sixth maintenance period	51.2 ± 20.2	55.9 ± 22.5	0.513
final maintenance period	46.8 ± 23.4	52.2 ± 21.0	0.594
Maximum AST	245.3 ± 167.4	377.8 ± 355.7	0.743
Maximum ALT	483.6 ± 298.6	693.4 ± 500.3	0.510

* $p < 0.05$ was considered statistically significant. † 6-MP dose intensity: the ratio of actual prescribed 6-MP dose by physician and the protocol dose. Abbreviations: ANC, absolute neutrophil count; 6-MP, 6-mercaptopurine; *NUDT15*, nucleoside diphosphate-linked moiety X-type motif 15; *ITPA*, inosine triphosphate pyrophosphatase.
